# Efficacy of omalizumab after discontinuation: a retrospective single-center observational study in children with severe asthma

**DOI:** 10.3389/falgy.2025.1529624

**Published:** 2025-03-07

**Authors:** Simone Foti Randazzese, Cecilia Lugarà, Francesca Galletta, Giovanni Pioggia, Giuseppe Crisafulli, Lucia Caminiti, Sebastiano Gangemi, Paolo Ruggeri, Sara Manti

**Affiliations:** ^1^ Pediatric Unit, Department of Human Pathology in Adult and Developmental Age “Gaetano Barresi”, University of Messina, Messina, Italy; ^2^ Institute for Biomedical Research and Innovation (IRIB), National Research Council of Italy (CNR), Messina, Italy; ^3^ School and Operative Unit of Allergy and Clinical Immunology, Department of Clinical and Experimental Medicine, University of Messina, Messina, Italy; ^4^ Pulmonology Unit, Department of Biomedical, Dental, Morphological and Functional Imaging Sciences (BIOMORF), University of Messina, Messina, Italy

**Keywords:** children, discontinuation, omalizumab, persistence of efficacy, severe asthma

## Abstract

**Introduction:**

Several trials documented safety and efficacy of omalizumab, but there are a few data about its effects after discontinuation. This study aims to evaluate the maintenance of efficacy of omalizumab in pediatric asthmatic patients one year after its suspension.

**Methods:**

A retrospective analysis was conducted on 17 subjects aged 6–18 years, divided into two groups: Group A (9 patients) who discontinued omalizumab after 18 months, and Group B (8 patients) who continued the therapy. Data on respiratory function (FEV1%), the number of exacerbations, need for hospitalizations, use of oral corticosteroids, and Asthma Control Test (ACT) scores were collected and analyzed at three time points: baseline (T0), after 18 months of treatment (T1), and 36 months (T2).

**Results:**

In Group A, significant differences were observed between T0 and T1, and T1 and T2, in FEV1% values, the number of exacerbations, the need for oral corticosteroids, and ACT scores. Group B showed significant differences in these parameters over time, with a notable reduction in exacerbations and improvement in ACT scores. The comparative analysis revealed that Group B had a higher number of exacerbations compared to Group A at T0 and greater use of oral cortico-steroids at T1. By T2, Group A had a higher ACT score than Group B at T0, whereas Group B showed higher ACT scores at T2 compared to Group A.

**Discussion:**

The study confirmed the efficacy and safety of omalizumab, with its benefits persisting one year after treatment discontinuation in terms of lung function, reduction in exacerbations, decreased need for oral corticosteroids, and improved quality of life. Further research is necessary.

## 1 Introduction

Omalizumab is a humanized recombinant monoclonal antibody approved by the Food and Drug Administration (FDA) and the European Medicines Agency (EMA) as add-on treatment for patients ≥6 years old with moderate-to-severe persistent allergic asthma and unsatisfactory response to inhaled corticosteroid (ICS), high serum immunoglobulin E (IgE) levels (30–1,500 IU/ml) and positive specific serum IgE to at least one aeroallergen (1–3). Furthermore, omalizumab is recommended for patients aged 12 years and above with chronic spontaneous urticaria (CSU), as well as for adults (aged 18 years and above) with nasal polyps (1–3). In addition, several studies are ongoing involving omalizumab as an add-on treatment to oral immunotherapy (OIT) or as monotherapy in food allergy (FA) (4). Indeed, on February 16, 2024, the FDA approved omalizumab for the reduction of allergic reactions, including anaphylaxis, that may occur with an accidental exposure to one or more foods in adults and children aged 1 year and older with FA (5).

Omalizumab binds to the Cε3 domain of the Fc region of circulating IgE, forming inert complexes that do not activate the complement system, thereby reducing serum IgE levels. It also inhibits the interaction between IgE and their high-affinity (FcεRI) and low-affinity (FcεRII/CD23) receptors, which are present on the membranes of mast cells, basophils, eosinophils, neutrophils, dendritic cells, and lymphocytes. This reduces the expression of these receptors and inhibits the release of inflammatory mediators (6–8). Additionally, omalizumab acts on IgE bound to B-cell receptors (BCRs). The synthesis of IgE is regulated by the interaction between the IgE-BCR complex and CD21 on B cells, which are induced to synthesize IgE by soluble CD23 (sCD23). The binding of omalizumab to the IgE-BCR complex prevents IgE synthesis and induces cellular apoptosis (9). Finally, omalizumab can enhance the antiviral response in patients with allergic asthma, particularly those with high serum IgE levels who are more susceptible to viral-induced exacerbations, especially from respiratory viruses like Rhinovirus and Influenza virus (10, 11). This effect is attributed to its action on plasmacytoid dendritic cells, which typically produce interferon (IFN), particularly IFN-α, following interactions between virions and Toll-Like Receptors (TLRs) expressed on their plasma membrane (12). Compared to healthy individuals, patients with allergic asthma exhibit increased expression of FcεRI on dendritic cells and elevated IgE levels, which correlates with a significant down-regulation in TLRs expression and a reduced production of IFN-α in response to viral infections (12). Therefore, treatment with omalizumab also improves the TLR-mediated antiviral response, enhancing IFN-α production by dendritic cells (12). The drug is administered as a subcutaneous injection. In asthmatic subjects, the dosage and frequency of administration are personalized for each patient, established by a nomogram based on the patient's weight (kg) and baseline total serum IgE levels (13, 14).

Several studies in the literature documented the efficacy and safety of omalizumab. Specifically, omalizumab showed its efficacy after 12–16 weeks of treatment improving the number of exacerbations and hospitalizations, the need for rescue therapy and the quality of life (QoL) of patients and their caregivers (15–20).

Despite the clear benefits of omalizumab in the management of severe asthma, nowadays, stopping therapy is still debatable, as well as the criteria for safely suspending treatment are not well-defined. Factors such as stable lung function, reduced exacerbation frequency, and the ability to maintain asthma control were considered, but standardized guidelines are lacking. The decision is further complicated by the limited data available in current literature regarding the persistence of omalizumab beneficial effects after discontinuation among adult and, particularly, pediatric patients (18, 21–28).

The aim of the following study was to assess the sustained efficacy of omalizumab therapy in pediatric patients with severe asthma one year after treatment discontinuation. Specifically, our evaluation focused on several key indicators of asthma control, including Forced Expiratory Volume in the 1st second (FEV1%), number of annual exacerbations, need for hospitalization and oral corticosteroid (OCS) use, and QoL.

## 2 Materials and methods

### 2.1 Study design

This study was a retrospective, single-center, observational analysis focusing on pediatric patients with severe asthma. We collected a range of demographic and clinical data, including FEV1% values, number of exacerbations, data about need for hospitalization and OCS, scores from the Asthma Control Test (ACT).

### 2.2 Subjects and eligibility criteria

The study population consisted of pediatric patients of both genders, aged 6–18 years old, affected by severe asthma. The following inclusion criteria were adopted: age major than 6 years old; confirmed diagnosis of severe asthma in agreement with the current guidelines (1, 29); therapy with omalizumab administered for at least 18 months; discontinuation of omalizumab treatment for at least one year. Patients with good control of the disease, in terms of FEV1% (≥90%), number of exacerbations (<3/year), need for hospitalization (0/year), need for OCS (0/year) and ACT score (≥20), were candidates for discontinuing treatment.

### 2.3 Data analysis

The data were analysed using Microsoft Excel version 2023 and the Statistical Package for Social Sciences (SPSS) software version 22.0. Data were considered statistically significant with a *p* value <.05. The continuous variables were categorized using descriptive statistics and expressed as mean ± standard deviation (SD). The ordinary variables were expressed as percentage. Fisher's exact test or the Pearson Chi-squared test (Pearson coefficient of correlation) for qualitative variables and the paired *t*-test for continuous variables were used.

### 2.4 Ethics

All study participants, along with their parents, received comprehensive information and provided written informed consent as part of the study protocol. The study protocol was designed and conducted in compliance with Good Clinical Practice (GCP) standards and adhered to the ethical principles outlined in the Declaration of Helsinki with successive amendments. The Local Ethics Committee confirmed that no ethical approval was required for this retrospective observational study.

## 3 Results

17 children and adolescents affected by severe asthma and treated with omalizumab were included in the final analysis. Based on the suspension of omalizumab, the enrolled population was stratified into two groups: Group A, including 9/17 (53%) patients who stopped omalizumab; Group B, including 8/17 (47%) patients who continued the treatment with omalizumab. The clinical and demographic features of the enrolled population are shown in Table 1.

**Table 1:** Demographic and clinical findings of the enrolled population (*n* = 17).

Children's characteristics	Group A (*n* = 9)	Group B (*n* = 8)
Gender, *n* (%)	Male, 6 (67)	Male, 4 (50)
	Female, 3 (33)	Female, 4 (50)
Race, *n* (%)	Caucasian, 8 (89)	Caucasian, 8 (100)
	Asian, 1 (11)	
Comorbidities, *n* (%)	AR, 6 (67)	AR, 7 (87.5)
	AD, 2 (22)	FA, 2 (25)
	EoE, 1 (11)	CSU and HT, 1 (12.5)
		PCOS, 1 (12.5)
Age at the diagnosis of asthma, mean (SD), years	7.5 (2.9)	6.8 (2.0)
Age at omalizumab initiation, mean (SD), years	10.6 (3.5)	11.0 (3.6)
Treatment duration, mean (SD), years	2.1 (0.9)	—
Age at the treatment discontinuation, mean (SD), years	12.5 (3.8)	—
Adverse drug reactions, *n* (%)	0	1 (12.5)
Discontinuation reasons, *n* (%)	Patients and/or caregivers request, 8 (89)	—
	Transfer abroad, 1 (11)	

AR, allergic rhinitis; AD, atopic dermatitis; CSU, chronic spontaneous urticaria; EoE, eosinophilic esophagitis; FA, food allergy; HT, Hashimoto thyroiditis; *n*, number; PCOS, polycystic ovary syndrome; SD, standard deviations.

Regarding Group A, 6/9 (67%) were male and 3/9 (33%) were female. 8/9 (89%) were Caucasian and 1/9 (11%) was Asian. 8/9 (89%) presented comorbidities: 6/9 (67%) allergic rhinitis (AR), 2/9 (22%) atopic dermatitis (AD) and 1/9 (11%) eosinophilic esophagitis (EoE). The mean age at the diagnosis of asthma was 7.5 ± 2.9 years old. The mean age at the start of omalizumab was 10.6 ± 3.5 years old, with a mean duration of therapy of 2.1 ± 0.9 years, and a mean age at the treatment discontinuation of 12.5 ± 3.8 years old. None of the patients developed adverse drug reactions (ADRs) due to omalizumab. All the patients continued therapy with ICS/Long-Acting Beta2-Agonist (LABA) during and post-treatment with omalizumab. 8/9 (89%) stopped omalizumab treatment according to patient and/or caregivers request, 1/9 (11%) suspended omalizumab for moving abroad. Before the suspension, the asthma control of the disease was evaluated according to the previously reported criteria. Regarding Group B, 4/8 (50%) were male and 4/8 (50%) were female. 8/8 (100%) were Caucasian. 8/8 (100%) presented comorbidities: 7/8 (87.5%) AR, 2/8 (25%) FA, 1/8 (12.5%) CSU and Hashimoto thyroiditis (HT) and 1/8 (12.5%) polycystic ovary syndrome (PCOS). The mean age at the diagnosis of asthma was 6.8 ± 2.0 years old, with a mean age at the start of the treatment with omalizumab of 11.0 ± 3.6 years old. 1/8 (12.5%) patient developed mild and transient headache because of omalizumab administration. All the patients continued therapy with ICS/LABA during treatment with omalizumab. No monoclonal antibodies were administered before omalizumab in both groups. The dosage and frequency of omalizumab administration were established by the nomogram, as previously stated.

Regarding Group A, the statistical analysis was conducted at baseline (T0), at 18 months of treatment (T1), considered a common reference for mid-treatment assessment, and one year after the suspension of omalizumab (T2). The results are reported in Table 2. The following changes were reported: in FEV1% values T0: 87.4 ± 7.8 vs. T1: 104.6 ± 13.8 (*p* = .005) vs. T2: 119.2 ± 7.1 (*p* = .012); in the number of exacerbations: T0: 5.9 ± 2.3 vs. T1: 3.5 ± 2.1 (*p* = .036) vs. T2: 1.8 ± 0.7 (*p* = .025); in the number of patients who needed hospitalization: T0: 67% vs. T1: 0% (*p* = .001) vs. T2: 11% (*p* = .332); in the number of patients who needed OCS: T0: 100% vs. T1: 11% (*p* = .000) vs. T2: 56% (*p* = .048); and in ACT score: T0: 14 ± 1.6 vs. T1: 21.7 ± 2.6 (*p* < .000) vs. T2: 18.2 ± 5.5 (*p* = .013).

**Table 2:** Results of the statistical analysis of Group A (*n* = 9).

Variables	T0	T1	*p*	T2	*p*
FEV1, mean (SD), %	87.4 (7.8)	104.6 (13.8)	=.005	119.2 (7.1)	=.012
Exacerbations, mean (SD), *n*/year	5.9 (2.3)	3.5 (2.1)	=.036	1.8 (0.7)	=.025
Need for hospitalization, *n* (%)	67	0	=.001	11	=.332
Need for OCS, *n* (%)	100	11	=.000	56	=.048
ACT score, mean (SD)	14 (1.6)	21.7 (2.6)	=.000	18.2 (5.5)	=.013

ACT, Asthma Control Test; FEV1%, Forced Expiratory Volume in the 1st second; *n*, number; OCS, oral corticosteroid; SD, standard deviations.

Regarding Group B, the statistical analysis was conducted at baseline (T0), at 18 months (T1) and at 36 months of treatment (T2). The results are reported in Table 3. The following changes were reported: in FEV1% values T0: 84.1 ± 10.6 vs. T1: 97.5 ± 12.7 (*p* = .044) vs. T2: 112.6 ± 13.3 (*p* = .035); in the number of exacerbations: T0: 9.1 ± 2.7 vs. T1: 4.7 ± 0.7 (*p* = .000) vs. T2: 2.0 ± 0.7 (*p* < .000); in the number of patients who needed hospitalization: T0: 87.5% vs. T1: 0% (*p* = .008) vs. T2: 11% (*p* = .148); in the number of patients who needed OCS: T0: 100% vs. T1: 62.5% (*p* = .059) vs. T2: 12.5% (*p* = .040); and in ACT score: T0: 12.2 ± 1.2 vs. T1: 19.5 ± 2.9 (*p* = .000) vs. T2: 22.6 ± 1.5 (*p* = .020).

**Table 3:** Results of the statistical analysis of Group B (*n* = 8).

Variables	T0	T1	*p*	T2	*P*
FEV1, mean (SD), %	84.1 (10.6)	97.5 (12.7)	=.044	112.6 (13.3)	=.035
Exacerbations, mean (SD), *n*/year	9.1 (2.7)	4.7 (0.7)	=.000	2.0 (0.7)	<.000
Need for hospitalization, *n* (%)	87.5	0	=.008	11	=.148
Need for OCS, *n* (%)	100	62.5	=.059	12.5	=.040
ACT score, mean (SD)	12.2 (1.2)	19.5 (2.9)	=.000	22.6 (1.5)	=.020

ACT, Asthma Control Test; FEV1%, Forced Expiratory Volume in the 1st second; *n*, number; OCS, oral corticosteroid; SD, standard deviations.

Finally, a comparative analysis between Group A and Group B was performed. The results are shown in Table 4. Specifically: FEV1%: *p* = .472 (T0), *p* = .286 (T1), *p* = .213 (T2); number of exacerbations: *p* = .017 (T0), *p* = .141 (T1), *p* = .529 (T2); number of patients who needed hospitalization: *p* = .342 (T0), *p* = .124 (T1), *p* = .362 (T2); number of patients who needed OCS: *p* = .002 (T1), *p* = .168 (T2); ACT score: *p* = .025 (T0), *p* = .132 (T1), *p* = .046 (T2).

**Table 4:** Results of the comparative analysis between Group A and Group B.

Variables	*p* T0	*p* T1	*p* T2
FEV1%	=.472	=.286	=.213
Number of annual exacerbations	=.017	=.141	=.529
Need for hospitalization	=.342	=.124	=.362
Need for OCS	=.500	=.002	=.168
ACT score	=.025	=.132	=.046

ACT, Asthma Control Test; FEV1%, Forced Expiratory Volume in the 1st second; OCS, oral corticosteroid.

## 4 Discussion

Children and adolescents with severe asthma face different challenges; their condition not only compromises their physical health, but also has a profound impact on their QoL, potentially hindering their daily activities, overall growth, and developmental progress (30, 31). The burden of severe asthma can lead to frequent absences from school, physical activity limitations, and psychological stress (31). Omalizumab is widely used as a long-term treatment for managing severe, persistent asthma, making it crucial to understand whether its therapeutic benefits endure after the treatment is discontinued, especially in pediatric patients.

Our study aimed to analyze whether stopping omalizumab treatment would lead to a deterioration in asthma control or if the clinical improvements achieved during the therapy could be sustained even without continued pharmacological intervention. This evaluation is particularly important as discontinuing biologic therapy represents a delicate step for patients, families, and healthcare providers. Understanding the persistence of treatment benefits could influence clinical decision-making regarding the long-term management strategies for pediatric subjects with severe asthma. Nopp et al. described the clinical and immunological state of 18 adult patients with severe allergic asthma 3 years after omalizumab was stopped. 12/18 (66.7%) patients reported improved or unchanged asthma control compared with ongoing treatment in terms of FEV1%, QoL at questionnaires and downregulation of basophil allergen sensitivity (22). These data were confirmed by additional studies. Humbert et al. conducted a real-life study to assess omalizumab treatment patterns in adult and pediatric asthmatic patients and describe asthma control at omalizumab initiation and discontinuation. 16,750 adults and 2,453 children started omalizumab, with a median treatment persistence before discontinuation of 51.2 months in adults and 53.7 months in children. Among adults who discontinued omalizumab while asthma was controlled, 70%, 39% and 24% remained controlled and did not resume omalizumab at 1, 2 and 3 years after suspension, respectively. These proportions were higher in children (76%, 44% and 33%, respectively). Over 2 years of follow-up after discontinuation, rate of hospitalizations for asthma (none before the suspension, 1.3% and 0.6% at 2 years in adults and children respectively) and use of OCS (20.0% and 20.2% before the stop, 33.3% and 24.6% at 2 years in adults and children respectively) remained stable (23). Recently, Ferraro et al. conducted a prospective study on a cohort of 20 pediatric subjects who discontinued omalizumab after at least 2 years of treatment. Patients were evaluated at T0 (when omalizumab was discontinued) and after 3 (T1), 6 (T2) and 12 (T3) months after the suspension in different items: number of asthma exacerbations, asthma control according to Global Initiative for Asthma (GINA), Composite Asthma Severity Index (CASI), and spirometry. Furthermore, the Pediatric Asthma Quality of Life Questionnaire (PAQLQ) was administered at T0 and T3. The study showed omalizumab's clinical and functional effect for at least 1 year after discontinuation; only one child resumed omalizumab for worsening asthma, suggesting that a minority of children with severe allergic asthma may depend on biological therapy (24). Regarding our study, we analyzed and statistically correlated 5 items (FEV1%, number of exacerbations, need for hospitalization, need for OCS, and ACT score) at baseline (T0), at 18 months (T1) and at 36 months (T2) in two pediatric cohorts of severe asthmatic patients treated with omalizumab: Group A, including 9 subjects who stopped omalizumab; Group B, including 8 subjects who continued the treatment. In Group A, we found a statistically significant correlation in FEV1% values (*p* = .005 and =.012), number of exacerbations (*p* = .036 and =.025), number of patients who needed OCS (*p* = .000 and =.048) and ACT score (*p* = .000 and =.013) between T0 and T1, and T1 and T2, respectively, demonstrating the persistence of omalizumab efficacy one year after its discontinuation. In Group B, a statistically relevant correlation was detected in FEV1% values (*p* = .044 and =.035), number of exacerbations (*p* = .000 and <.000) and ACT score (*p* = .000 and =.020) between T0 and T1, and T1 and T2, respectively, confirming current literature data on the efficacy of omalizumab in pediatric subjects with severe asthma. Additionally, the comparative analysis performed between the two groups at T0, T1 and T2, did not document a statistically significant difference in FEV1% values and in the number of patients who required hospitalization. Patients in Group B showed a higher number of exacerbations at T0 (*p* = .017) and greater use of OCS (*p* = .002) at T1 compared to Group A. Finally, patients in Group A showed higher ACT scores than Group B at T0 (*p* = .025), whereas Group B showed higher ACT scores at T2 compared to Group A (*p* = .046).

In their retrospective analysis, Silver et al. showed that the most common reasons of discontinuation were lack of symptoms control, exacerbations, cost, and patient re-quest, highlighting the complexity of care for this group of subjects and the need for assessment the reasons for discontinuation, including both clinical and non-clinical factors (25). Therefore, establishing the criteria for discontinuation remains an essential aspect, as suggested by Kupryś-Lipińska et al. (26). Their findings highlighted that the decision to stop omalizumab should be individual and based on benefits and risks, especially in patients with a long history of severe asthma, treated with high doses of OCS before the introduction of omalizumab, near-fatal asthma events and/or worsening of asthma during previous trials of omalizumab discontinuation (26). In our study, all patients discontinued omalizumab after a strict assessment of asthma control, based on evaluation of respiratory function, number of asthma exacerbations, need for hospitalization, need for OCS, and ACT score, as previously de-scribed. However, it is desirable to establish consistent criteria for discontinuing treatment in patients receiving omalizumab or other monoclonal antibodies.

Another challenge is establishing the duration of the omalizumab efficacy once suspended. *in vitro* studies showed that IgE production decreases throughout treatment, reaching a new equilibrium after about 5 years. It was suggested that IgE production could increase slowly after discontinuation, returning to baseline after 15 years, meaning patients would not need omalizumab indefinitely (32). Vennera et al. conducted an open, prospective study evaluating 49 adult patients who voluntarily agreed to stop omalizumab after 6 years of treatment. A total of 19 subjects (38.8%) developed asthma exacerbations: 12 patients relapsed in the first year of follow-up, and 7 within 13 and 48 months vs. 30 patients (61.2%) who did not relapse. These results suggest that the efficacy of omalizumab after 6 years of treatment may persist for at least 4 years after discontinuation of therapy (28). Regarding our study, all the patients presented satisfactory asthma control one year after the suspension. Nobody needs to restart treatment with omalizumab or other monoclonal antibodies. Still, a continuous follow-up is necessary to determine the optimal therapy duration and maintain its efficacy at the stop in long-term studies.

## 5 Conclusion

Our study provides additional real-world evidence on the maintenance of omalizumab efficacy following treatment discontinuation, contributing to the broader understanding of its impact on asthma management. Despite the limited sample size, our retrospectively collected data confirmed the efficacy and safety of omalizumab, demonstrating sustained benefits in key clinical parameters, including FEV1%, annual exacerbation rate, need for OCS, and QoL. However, our study was not designed to establish definitive criteria for determining when omalizumab should be discontinued or continued. Given the potential for variability in patient response, individuals who discontinue treatment should be closely monitored to ensure sustained asthma control. While our findings contribute to the ongoing discussion on omalizumab discontinuation, further prospective, long-term studies with larger cohorts are essential to develop evidence-based guidelines for treatment cessation once optimal asthma control has been achieved.

## Data Availability

The raw data supporting the conclusions of this article will be made available by the authors, without undue reservation.
